# Design and Analysis of Minimum-Weighted Connected Capacitated Vertex Cover Algorithms for Link Monitoring in IoT-Enabled WSNs

**DOI:** 10.3390/s26092752

**Published:** 2026-04-29

**Authors:** Miray Kol, Ege Erberk Uslu, Zuleyha Akusta Dagdeviren, Orhan Dagdeviren

**Affiliations:** Department of Computer Engineering, Ege University, Izmir 35100, Turkey; miray.kol@ege.edu.tr (M.K.); ege.erberk.uslu@ege.edu.tr (E.E.U.); orhan.dagdeviren@ege.edu.tr (O.D.)

**Keywords:** internet of things, wireless sensor networks, link monitoring, network security, graph theory

## Abstract

Wireless sensor networks (WSNs) are the backbone of IoT-enabled smart manufacturing, environmental monitoring, and industrial automation. However, their broadcast nature makes communication links vulnerable to eavesdropping, routing manipulation, and denial-of-service attacks. Strategically placing monitor nodes to check each link is an effective approach to protect against attacks, but energy, connectivity, and capacity constraints should be considered while picking monitor nodes. In this paper, we tackle the Minimum-Weighted Connected Capacitated Vertex Cover (MWCCVC) problem, which minimizes monitoring costs, ensures backbone connectivity, and adheres to per-node capacity constraints. Unlike prior works that consider weighted vertex cover, connectivity constraints, or capacitated variants separately, the proposed MWCCVC model jointly integrates all three dimensions within a single vertex cover-based monitoring framework. We first provide a Branch-and-Bound (B&B) solver with linear programming relaxation bounds and constraint-based pruning strategies that produces optimum solutions. Three constructive greedy heuristics (GD, GR, GW) and two hybrid genetic algorithms (HGA, HGA-v2) that combine parameterized greedy decoders with evolutionary search are proposed; all methods guarantee full edge coverage, induced-subgraph connectivity, and max-flow-validated capacity feasibility. Tests on 130 small, 160 medium, and 19 large benchmark instances show that HGA matches B&B optima on every small instance, beats the time-limited B&B by 6.6% on medium instances, where the percentage is computed based on the relative difference in average total weight with respect to B&B, and stays the best on large graphs with up to 1000 nodes. The HGA-v2 tries to balance the quality and speed, with only a 3.1% difference at 10× faster execution.

## 1. Introduction

Wireless Sensor Networks (WSNs) are a fundamental component of the Internet of Things (IoT) physical systems, enabling data collection across diverse environments [1,2,3,4]. However, the broadcast nature of wireless communication exposes links to security threats such as eavesdropping, routing manipulation, and denial-of-service attacks. A practical defense is the strategic placement of monitor nodes that inspect traffic on surrounding links. However, activating monitor nodes incurs energy consumption, which is a critical constraint in resource-limited WSNs. Therefore, it is essential to minimize monitoring cost while ensuring complete link coverage and maintaining connectivity among selected monitors.

Monitoring problems in WSNs can be naturally formulated within a graph-theoretic framework. To this end, the network is modeled as an undirected graph G=(V,E), in which the vertex set represents sensor nodes and the edge set corresponds to communication links. Under this representation, the monitoring task can be associated with the vertex cover (VC) concept. More precisely, a VC is a subset of vertices such that every edge in the graph has at least one endpoint contained in this subset. Hence, activating the vertices in the cover set as observer nodes guarantees that each communication link is monitored by at least one active node. Determining a VC of minimum cardinality, however, gives rise to the minimum vertex cover (MVC) problem, which is a well-known NP-hard combinatorial optimization problem.

The traditional MVC formulation is insufficient for WSN monitoring problems because it does not take into account the varying energy costs associated with activating different sensor nodes, the limited number of connections each node can physically monitor, and the necessity for the monitoring nodes to form a unified communication infrastructure. Addressing all these requirements simultaneously makes the problem even more complex and restricts the solution options. When the selected observer nodes need to form a connected subgraph to transmit monitoring data, the problem transforms into the connected vertex cover (CVC) problem. If the nodes have weights representing the monitoring cost, remaining energy, or placement expenses, the goal is to select the observer nodes with the lowest total weight. On the other hand, if the observer nodes can only monitor a limited number of communication links due to energy or computational constraints, the problem transforms into a capacitated vertex cover (CapVC) problem. The minimum-weighted capacitated connected vertex cover (MWCCVC) problem, which addresses node weights, connectivity requirements, and monitoring capacity constraints together, is the most comprehensive model that encompasses all these requirements.

This study introduces a novel formulation of the MWCCVC problem for link monitoring in IoT-enabled WSNs. Unlike prior studies that address weighted vertex cover, connectivity constraints, or capacitated variants separately, the proposed model jointly incorporates weight, connectivity, and capacity within a single unified framework. To the best of our knowledge, this is the first study in the literature to consider these three constraints simultaneously in a vertex cover-based monitoring setting. In methodological terms, this study develops three complementary greedy strategies, namely degree-based, ratio-based, and weight-based, and further proposes two hybrid genetic algorithms, HGA and HGA-v2, to obtain high-quality solutions efficiently.

In this study, we tackle the MWCCVC problem in IoT-enabled WSNs for energy-efficient link monitoring. The contributions of this article are summarized as follows:

1. We formulate the MWCCVC problem for WSNs modeled as undirected graphs. The formulation integrates monitoring cost, connectivity requirements of the monitoring backbone, and per-node capacity constraints within a unified optimization model.
2. We develop a Branch-and-Bound (B&B) algorithm that utilizes linear programming (LP) relaxation bounds and constraint-based pruning strategies to obtain reference optimal solutions.
3. We design three constructive greedy heuristics including Greedy Degree (GD), Greedy Ratio (GR), and Greedy Weight (GW). Each heuristic employs different node-selection and edge-ordering strategies. These methods also generate high-quality seed solutions for the proposed evolutionary algorithms.
4. We propose two hybrid genetic algorithms named HGA and HGA-v2. These algorithms combine parameterized greedy decoders with evolutionary optimization over decoder parameters and node-level random keys. All candidate solutions enforce full edge coverage, induced-subgraph connectivity, and exact capacity feasibility through a max-flow-based validation procedure.
5. We perform extensive computational experiments on benchmark datasets that include 130 small-scale instances, 160 medium-scale instances, and 19 large-scale instances. The results show that our proposed hybrid algorithms obtain optimal solutions for all small-scale instances. Furthermore, for medium-scale and large-scale graphs, we observe that the proposed methods produce favorable results in terms of both solution quality and computational efficiency.

The rest of the paper is structured as follows. A review of the literature on VC variants and monitoring problems is provided in Section 2. The system model together with the formal problem definition is presented in Section 3. Section 4 introduces the B&B solver used as a reference method. The proposed greedy and hybrid genetic algorithms are described in Section 5. Details of the experimental design are given in Section 6, and the corresponding results are presented in Section 7. The implications of these results are examined in Section 8, whereas concluding remarks are offered in Section 9.

## 2. Related Work

The problem of monitoring communication links in IoT and WSN environments has been widely studied using graph-theoretic optimization techniques [5,6]. Nodes represent physical devices and edges represent wireless communication links [3,7,8], and the objective is to select a subset of nodes capable of observing every link in the network [2,5]. This section reviews prior work related to VC, its weighted and connected extensions, capacitated formulations, and the algorithmic strategies developed for these problems. A comprehensive classification is provided in Table 1. To the best of our knowledge, no prior study addresses the MWCCVC problem.

### 2.1. Vertex Cover

The basic VC problem has garnered significant research interest under various algorithmic approaches. For a graph G=(V,E), the goal is to select a subset S⊆V such that at least one endpoint of every edge is covered [9], meaning that each communication link is observed by at least one monitoring node. VC-based monitoring is widely used in fault detection, traffic monitoring, and connection verification [1,10].

Local search methods include the two-stage exchange local search algorithm named NuMVC by Cai et al. [11], noise-based extensions for large-scale graphs by Ma et al. [12], and a theoretical analysis of iterative local search on sparse samples by Witt [13]. Constructive heuristics include the isolation heuristic [14] and the centrality-based Malatya VC [10]. Among evolutionary approaches, Wu et al. proposed a snowdrift-game-based memetic algorithm with strong convergence properties [15]. More recently, learning-based approaches have also been explored. Lazzarinetti  et al. [16] introduced an attention-based mechanism that leverages both local and global adjacency information to obtain approximate MVC solutions on complex networks, thereby demonstrating the potential of neural methods to complement traditional heuristics. Theoretical work covers covering constraints on hypergraphs [17], hard capacity constraints [18], partial VC in bipartite graphs [19], and LP relaxation lower bounds [20]. In distributed WSN applications, Kavalci et al. [21] and Yigit et al. [22] evaluated self-stabilizing and distributed VC algorithms, while Akram and Ugurlu proposed a localized scalable algorithm [23]. Despite this extensive literature, classical VC formulations do not account for node activation costs, capacity constraints, or backbone connectivity.

**Table 1:** Summary of related work on vertex cover variants.

Ref.	Problem	Network	Heur./Greedy	Local Search	Dist./Approx.	Metaheur.	Exact/B&B	VC/Cover	Weight/Cost	Runtime	Convergence	Key Novelty
[5]	Multi ^†^	Undir.	✓	–	–	–	–	✓	–	✓	–	Multi-variant greedy for VC, MWVC, CapVC, CVC
[14]	VC	General	✓	–	–	–	–	✓	–	–	–	Isolation heuristic for unweighted min VC
[11]	VC	General	–	✓	–	–	–	✓	–	✓	–	Two-stage exchange local search for MVC
[15]	VC	General	–	–	–	✓	–	✓	–	–	✓	Snowdrift game + memetic algorithm
[21]	VC	General	–	–	✓	–	–	–	–	–	–	Comparison of distributed VC algorithms
[23]	VC	General	–	–	✓	–	–	–	–	–	–	Localized distributed VC algorithm
[10]	MVC	General	✓	–	–	–	–	✓	–	–	–	Centrality-driven greedy (Malatya VC)
[16]	MVC	General	–	–	–	✓	–	✓	–	–	–	Attention-based GNN for MVC on complex networks
[24]	WVC	General	–	–	✓	–	–	–	✓	✓	–	Primal-dual WVC solver
[25]	WVC	Massive	–	✓	–	–	–	–	✓	–	–	Local search algorithm with reduction rules
[26]	MWVC	Massive	–	–	–	✓	–	–	✓	✓	–	Accelerated local search for MWVC
[27]	MWVC	General	–	✓	–	✓	–	–	✓	–	–	MS-ITS for MWVC
[28]	MWVC	General	–	–	–	✓	–	–	✓	–	–	Fixed set search (GRASP)
[29]	MWVC	Massive	✓	–	–	✓	–	✓	✓	✓	–	GNN-based heuristic with reduction rules for MWVC
[30]	CVC	4-Regular	–	–	✓	–	–	✓	–	–	–	Approx. for CVC on 4-regular graphs
[31]	CVC	General	✓	✓	–	–	–	✓	–	✓	–	Two-stage greedy local-search for CVC
[32]	WCVC	Unit disk	–	–	✓	–	–	–	✓	–	–	PTAS for WCVC on UDGs
[1]	WCVC	Undir.	–	–	–	✓	–	✓	✓	–	✓	Hybrid GA for WCVC
[33]	WCVC	Undir.	✓	–	–	✓	–	✓	✓	✓	–	PBIG for VC-based monitoring
[34]	CapVC	General	–	–	✓	–	–	✓	–	–	–	CapVC evaluation for WSN security
[35]	CapVC	General	✓	–	✓	–	–	✓	–	–	✓	Self-stabilizing CapVC, O(n) steps
[7]	CapVC	General	–	–	–	–	✓	–	–	✓	–	Tree-capacity structure model
[36]	WCapVC	General	–	–	✓	–	–	–	✓	–	–	Dynamic weighted VC, soft capacities
[37]	CapVC	General	–	–	✓	–	–	✓	–	–	–	FPT (1+o(1))-approximation via treewidth
[38]	Deploy.	WSN	–	–	–	✓	–	✓	–	–	–	GA-PSO hybrid for WSN deployment
[39]	Cluster.	WSN	–	–	–	✓	–	–	–	–	–	Quantum-inspired PSO for clustering
[40]	Deploy.	WSN	–	–	–	–	–	–	–	–	–	Review of coverage and energy-efficient WSN deployment
[41]	VC	WSN	✓	–	–	–	–	✓	–	–	–	BFS tree + VC for routing
[42]	MVC	Massive	–	–	–	✓	–	✓	–	✓	–	Edge-age local search for MVC
[43]	MVC	Large	–	–	–	✓	–	✓	–	✓	–	3-improvement with perturbation
[44]	MVC	General	–	–	–	✓	–	✓	–	–	–	Membrane evolutionary algorithm
[45]	MVC	General	–	✓	–	✓	–	✓	–	–	✓	Hybrid CRO + BFS
Ours	MWCCVC	Undir.	✓	–	–	✓	✓	✓	✓	✓	✓	First MWCCVC on general undirected graphs

^†^ Covers VC, MWVC, CapVC, and CVC variants simultaneously.   ✓ = present;   – = not applicable or not reported.

### 2.2. Weighted Vertex Cover

The Minimum-Weighted Vertex Cover (MWVC) problem generalizes VC by associating a weight with each node, representing energy level, monitoring cost, or computational capacity, with the goal of minimizing the total weight of the selected set. Approaches include the primal–dual solver by Xu et al. [24], the fast list heuristic by Shimizu et al. [46], the local search algorithm with reduction rules, namely NuMWVC [25], dynamic strategies for large-scale graphs [47], the multi-start iterative tabu search (MS-ITS) [27], chemical reaction optimization (CRO) [48], accelerated local search [26], a binary evolutionary method [49], and a greedy randomized adaptive search procedure (GRASP) [28]. In parallel with these classical strategies, learning-based heuristics have also emerged. Langedal et al. [29] proposed a graph neural network (GNN) heuristic that integrates learned node representations with reduction rules and achieved competitive results on large-scale instances. Their study further illustrated that learning-based models can complement reduction-based exact solvers for the weighted variant. Although effective, none of these methods enforce connectivity among selected nodes, limiting their applicability for WSN backbone construction.

### 2.3. Connected Vertex Cover and Dominating Set

The CVC problem additionally requires the selected VC to form a connected subgraph [1,32], which is essential for creating a virtual backbone in WSNs. Algorithmic approaches include approximation algorithms for 4-regular graphs [30], parameterized complexity studies [50], and a two-phase greedy local search [31]. The closely related connected dominating set (CDS) has been studied by Du et al. via fixed-coefficient approximations [51,52], by Dagdeviren et al. via population-based algorithms [53], by Potluri and Singh via hybrid metaheuristics [54], and by Dahmri and Bouamama via nondominated sorting genetic algorithm (NSGA-II)  [55]. However, none of these works jointly address node weights and per-node capacity constraints.

### 2.4. Weighted Connected Vertex Cover

The Weighted Connected Vertex Cover (WCVC) problem extends CVC with node weights, minimizing total cost while maintaining connectivity [1]. Early work focused on geometric models: Fan et al. proposed a Polynomial-Time Approximation Scheme (PTAS) for unit disk graphs (UDGs) [32], and Wang et al. developed a PTAS for minimum-weighted CVC $P_{3}$ on UDGs [56]. For general undirected graphs, Dagdeviren proposed an HGA for WCVC [1], greedy methods for the minimum-weighted connected vertex cover (MWCVC) [1], and a population-based iterated greedy (PBIG) algorithm for connection monitoring [33]. These studies are the closest to our model but do not include per-node capacity constraints.

### 2.5. Capacitated Vertex Cover and Self-Stabilizing Approaches

The CapVC formulation imposes a capacity constraint c(v) on each node, addressing the fact that sensor nodes have limited processing and communication capacity that restricts the number of edges they can monitor [57,58], thereby preventing overloaded hubs and distributing workload more evenly [34,59,60]. Weighted Capacitated Vertex Cover (WCapVC) additionally incorporates energy awareness [5,36]. Yigit et al. evaluated CapVC algorithms for WSN security [34] and proposed the first distributed self-stabilizing CapVC with O(n) stabilization steps [35]. Delbot et al. proposed a distributed self-stabilizing CVC algorithm with an approximation ratio of 2 [61]. Other contributions include a tree-capacity model [7], a dynamic method for soft capacities [36], a hybrid Genetic Algorithm–Particle Swarm Optimization (GA-PSO) framework [38], and a quantum-inspired PSO method [39]. From a parameterized complexity perspective, Chu and Lin [37] recently developed FPT approximation algorithms for the capacitated vertex cover problem based on treewidth, providing (1+o(1))-approximation guarantees and highlighting the intrinsic algorithmic difficulty introduced by capacity constraints. Beyond algorithmic studies, the practical relevance of energy-aware monitor placement in WSNs has been further emphasized by Anusuya  et al. [40], who provided a systematic review of sensor node deployment strategies and confirmed that coverage and energy efficiency remain the principal optimization objectives in modern WSN design. These objectives directly motivate the weighted and capacitated formulation adopted in this work. However, none of these studies simultaneously combine weight minimization, spine connectivity, and capacity constraints.

## 3. System Model and Problem Formulation

### 3.1. System Model

**Figure 1:**
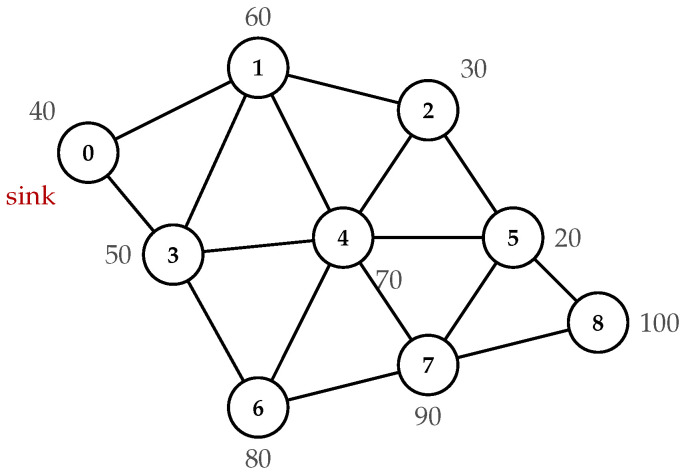
A sample WSN modeled as an undirected graph G=(V,E,w).

The IoT-enabled WSN is modeled as a node-weighted undirected graph G=(V,E,w), where *V* is the set of *n* sensor nodes, *E* is the set of *m* communication links, and $w:V\to R^{+}$ assigns an activation cost to each node. Each node v∈V is associated with a weight w(v), representing its energy-related activation cost. In this study, weights are derived from residual battery levels, capturing the heterogeneous energy distribution in real WSN deployments. Nodes located closer to the network core typically relay more traffic and thus deplete energy faster than peripheral nodes, leading to non-uniform residual energy levels. To reflect this, nodes with lower residual energy are assigned higher weights, discouraging their selection, while energy-rich nodes are assigned lower weights and are preferred. Consequently, minimizing $\sum_{v\in C}w\left(v\right)$ promotes energy-aware monitoring and helps extend the overall network lifetime. We assume a static topology during algorithm execution for tractability. Although real IoT-enabled WSNs may be dynamic, the static assumption is commonly used in optimization-based formulations and serves as a baseline. Figure 1 illustrates a sample network instance. In the figure, the unique identifier of each node is shown inside the corresponding node, whereas the associated node weight is given next to it. Node 0 is designated as the sink node. This example is provided for illustrative purposes only and does not correspond to the benchmark instances used in the experimental evaluation.

To illustrate the practical relevance of the MWCCVC formulation, consider an industrial manufacturing plant where n=200 battery-powered sensor nodes monitor temperature, vibration, and pressure across production lines. Each sensor communicates with its direct neighbors, and nodes have heterogeneous remaining energy levels reflected in the weight function w(v). The plant operator requires that every wireless link be inspected for anomalous traffic (edge coverage), that monitoring alerts reach the central supervisory control and data acquisition system through a connected relay path (connectivity), and that no single sensor is overloaded with more than K=15 simultaneous link inspections (capacity). In this setting, MWCCVC directly models the operator’s requirements, and the proposed algorithms select the energy-optimal monitor subset.

The weight w(v) represents the monitoring activation cost of node *v*. In practice, the most common interpretation is the reciprocal of residual battery energy. Under this interpretation, nodes with lower remaining energy receive higher weights, discouraging their selection and thereby prolonging the network lifetime. Alternative interpretations include deployment cost, hardware overhead, or risk exposure. The optimization objective minimizes the aggregate activation cost of all selected monitors, yielding an energy-balanced backbone.

Each sensor node has finite processing and energy resources that limit the number of links it can simultaneously monitor. This constraint is captured by a uniform per-node capacity $K\in Z^{+}$:

(1) cap(v)=K,∀v∈V.

The parameter *K* prevents monitors from being overloaded and distributes the workload evenly across the backbone.

A link e=(u,v)∈E is monitored if at least one endpoint belongs to the monitor set and *e* is explicitly assigned to that endpoint. This endpoint-assignment model reflects the physical reality that a monitor inspects packets on a specific subset of its incident links. The assignment must respect the capacity constraint.

During algorithm execution, nodes are classified as BLACK, which denotes a selected monitor, GRAY, which denotes a non-monitor with at least one BLACK neighbor, or WHITE, which denotes a node with no BLACK neighbor, and RED if a WHITE node is identified as a priority selection candidate. This coloring is an algorithmic bookkeeping device and does not correspond to optimization decision variables. We assume a static topology during algorithm execution [4] and that the sink has global graph information. Additionally, the sink node is not explicitly enforced as part of the monitoring set in the proposed formulation. However, in practical deployments, the sink can be incorporated as a mandatory node if required by the application.

### 3.2. MWCCVC Problem Formulation

The MWCCVC problem is defined on an undirected graph G=(V,E). For each vertex v∈V, let N(v)={u∈V:(u,v)∈E} denote the open neighborhood of *v*, i.e., the set of vertices adjacent to *v*. The closed neighborhood of *v* is defined as N[v]=N(v)∪{v}. Moreover, δ(v) denotes the set of edges incident to vertex *v*. Let C⊆V denote the set of selected vertices, referred to as monitors or the cover set. The subgraph induced by *C* is represented by G[C].

To model the problem, we introduce a binary decision variable $x_{v}\in \left\{0,1\right\}$ for each vertex v∈V. The variable $x_{v}$ takes value 1 if vertex *v* is selected as a monitor (i.e., v∈C), and 0 otherwise. In addition, for each edge e∈E and each endpoint v∈e, we define a binary variable $y_{e,v}\in \left\{0,1\right\}$. The variable $y_{e,v}$ equals 1 if edge *e* is assigned to vertex *v* for monitoring, and 0 otherwise.

The objective is to minimize the aggregate monitoring activation cost of all selected monitors:

(2) $$\left(\mathrm{MWCCVC}\right)\;\min\sum_{v\in V}w\left(v\right)\;x_{v}.$$

Since weights are positive, the formulation incentivizes selecting as few and as inexpensive monitors as possible. When weights represent reciprocal residual energy, minimizing (2) yields an energy-balanced backbone that prolongs network lifetime by avoiding the selection of energy-depleted nodes.

Every communication link must be observed by at least one monitor. This is the defining property of a VC:

(3) $$x_{u}+x_{v}\geq 1,\;\forall \left(u,v\right)\in E.$$

Constraint (3) guarantees that every data transmission between neighboring sensors is observed by at least one monitor node.

Each edge is assigned to exactly one of its selected endpoints for monitoring:

(4) $$\sum_{v\in e}y_{e,v}=1,\;\forall e\in E.$$

The equality in (4) prevents duplicate monitoring of the same link by both endpoints, freeing capacity for other links and creating unambiguous monitoring accountability.

An edge can only be assigned to a selected monitor:

(5) $$y_{e,v}\leq x_{v},\;\forall e\in E,\;\forall v\in e.$$

This coupling constraint ensures that only active monitors bear monitoring responsibilities.

Each monitor can be assigned at most *K* edges, reflecting the finite processing and energy resources of sensor nodes:

(6) $$\sum_{e\in \delta (v)}y_{e,v}\leq K\cdot x_{v},\;\forall v\in V.$$

The right-hand side collapses to zero when $x_{v}=0$, ensuring that non-monitors bear no assignment load.

The induced monitor subgraph must be connected to enable multi-hop relay of monitoring observations to the sink:

(7) $$G\left[C\right]\;\mathrm{is}\;\mathrm{connected},\;C=\left\{v\in V:x_{v}=1\right\}.$$

Constraint (7) ensures that monitors can communicate with each other and relay security alerts to the central sink through paths that lie entirely within the monitor backbone. In the proposed algorithms, connectivity is enforced via a repair procedure that inserts bridge vertices along weight-aware shortest paths between disconnected components.

The binary nature of the decision variables completes the formulation:

(8) $$x_{v}\in \left\{0,1\right\},\;\forall v\in V;\;y_{e,v}\in \left\{0,1\right\},\;\forall e\in E,\;v\in e.$$

The MWCCVC problem is NP-hard since both classical minimum VC [62] and connected VC [30] are special cases obtainable by relaxing the capacity and connectivity constraints, respectively. The simultaneous enforcement of all three constraint families, namely coverage (3), connectivity (7), and capacity (6), makes MWCCVC strictly harder than any of its individual components. This motivates the development of both exact (B&B) as described in Section 4 and heuristic/metaheuristic solution methods as presented in Section 5.

## 4. Proposed Branch-and-Bound Reference Solver

To obtain reference solutions, we develop a B&B algorithm that systematically explores the solution space via recursive partitioning and bound-based pruning. The algorithm maintains a search tree where each node corresponds to a partial assignment of $\left\{x_{v}\right\}$. At the root, all variables are unassigned; branching creates two children: $x_{v}\;=\;1$ and $x_{v}\;=\;0$. Expansion follows a best-first strategy using the smallest lower bound.

The branching variable is selected according to the most fractional rule in the LP relaxation. Specifically, the variable whose value is closest to 0.5 is chosen, with ties broken by the highest weighted degree. Before branching, several variable-fixing rules are applied to reduce the search space, including forced inclusion when an edge endpoint is excluded, degree-based fixing, and connectivity propagation for bridge vertices.

The LP relaxation of the formulation provides the lower bound, while connectivity requirements are handled through lazy subtour elimination cuts. An initial upper bound is obtained from the GR heuristic solution (will be explained in the following section) and is improved whenever the algorithm identifies a feasible integer solution during the search. Capacity feasibility is checked by solving a maximum-flow problem on a corresponding bipartite assignment network. Within the Branch-and-Bound procedure, a node is discarded if its bound is worse than the current incumbent value, if the LP relaxation is infeasible, or if the max-flow test indicates a violation of capacity constraints. The overall framework is described in Algorithm 1, where the search tree is explored by branching on fractional variables while maintaining the best feasible solution found so far.
**Algorithm 1:** Branch and Bound for MWCCVC.
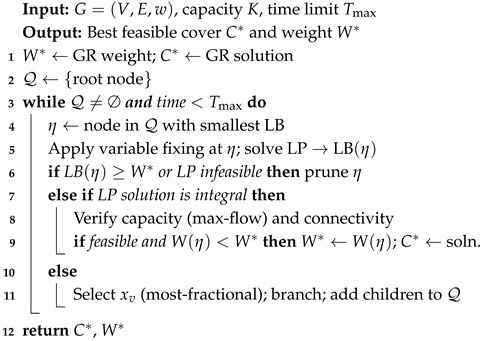


## 5. Proposed Heuristic and Metaheuristic Algorithms

### 5.1. Greedy Heuristic Strategies

We define three greedy heuristics that differ in their node-selection criterion. Each heuristic begins with all nodes colored WHITE. When a node *v* is selected, it is assigned the color BLACK, and its WHITE neighbors are changed to GRAY. The heuristics are

Greedy Degree (GCCVC) selects the node-maximizing uncovered degree:

(9) $$v^{*}=\arg\max_{v\in V_{\mathrm{cand}}}\left|N\left(v,\mathrm{WHITE}\right)\right|$$

with ties broken by minimum weight w(v). The rationale is that selecting high-degree nodes first reduces uncovered edges most rapidly, producing compact covers. Once a node is selected, its uncovered incident edges are sorted by decreasing the uncovered degree of the neighboring endpoint, then by increasing the neighbor weight, and are assigned to the selected node until its residual capacity is exhausted.

Greedy Ratio (GRCCVC) selects the node-minimizing cost-to-coverage ratio:

(10) $$v^{*}=\arg\min_{v\in V_{\mathrm{cand}}}\frac{w(v)}{\sum_{u\in N(v,\mathrm{WHITE})}w\left(u\right)}$$

with ties broken by minimum weight w(v). The numerator represents the direct cost of activating node *v*, while the denominator captures the aggregate weight of uncovered neighbors that would transition to GRAY status. The ratio criterion prioritizes nodes that provide maximum coverage benefit per unit cost, which is particularly effective under capacity constraints since each node can only cover a limited number of edges. This criterion adapts the ratio-based strategy originally developed for CDS [53] to the VC setting. Empirically, GRCCVC produces the lightest covers among all three greedy heuristics across all tested instances.

Greedy Weight (GWCCVC) selects the minimum-weight node:

(11) $$v^{*}=\arg\min_{v\in V_{\mathrm{cand}}}w\left(v\right)$$

with ties broken by minimum weight w(v). The rationale is that selecting the nodes with the highest uncovered degree first reduces uncovered edges most rapidly, producing compact covers. Once a node is selected, its uncovered incident edges are sorted by decreasing uncovered degree of the neighboring endpoint, then by increasing neighbor weight, and assigned to the selected node until its residual capacity is exhausted. The general greedy construction procedure is presented in Algorithm 2.
**Algorithm 2:** Greedy MWCCVC Construction.
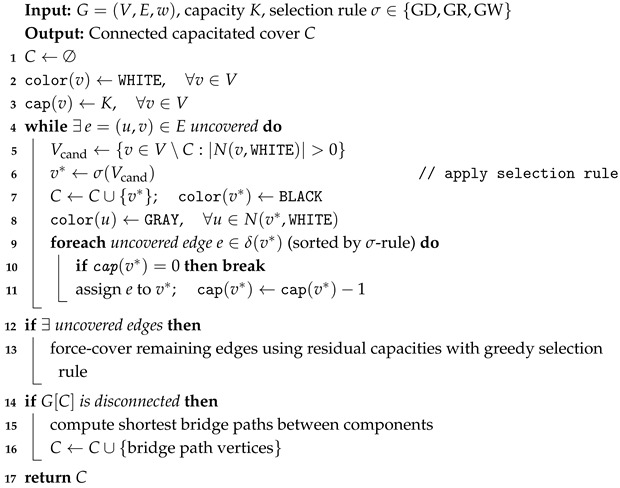


After the main constructive loop, uncovered edges may remain if selected monitors have exhausted their capacity before all incident edges could be assigned. In this case, a force-cover stage iterates over the remaining uncovered edges and assigns each to the endpoint with the largest residual capacity.

**Figure 2:**
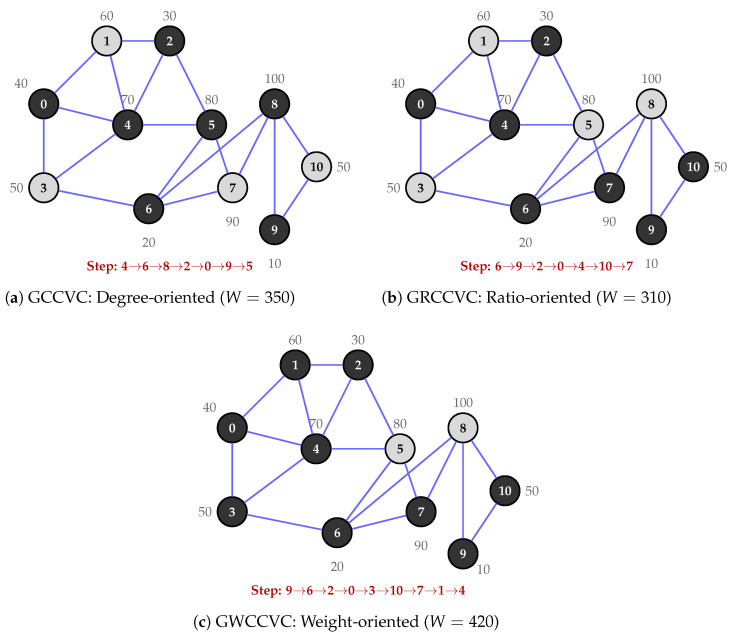
Example of MWCCVC solutions produced by the three greedy heuristics on a sample topology.

If the induced subgraph G[C] is disconnected after the constructive phase, a connectivity repair procedure is invoked. The procedure identifies connected components of G[C] and computes the shortest bridge paths between components in the original graph *G*. The vertices along these bridge paths are added to the cover, and their incident uncovered edges are assigned respecting capacity constraints. An illustrative example comparing these three strategies on a sample topology is shown in Figure 2, where the capacity limit is (K=5). The GCCVC selects node 4 first due to its highest uncovered degree (5 neighbors), then proceeds to cover the remaining edges through nodes 6, 8, and 2. GRCCVC begins with node 6 whose ratio 20/(50+80+90+100)=0.06 is minimal. GWCCVC starts with the lightest node 9 (W=10). Among these, GRCCVC produces the lowest VC total weight for the MWCCVC problem (W=310).

### 5.2. Hybrid Genetic Algorithm (HGA)

The proposed HGA is a population-based metaheuristic that represents solutions indirectly using chromosomes that encode parameters for a decoder. Rather than directly encoding a binary vector indicating which nodes belong to the cover, each chromosome consists of two parts: a six-dimensional continuous parameter vector $\theta =\left(a,b,c,d,e,\epsilon \right)\in {\left[0,3\right]}^{6}$ that controls the behavior of a greedy decoder, and a node-wise random-key map $r\in {\left[0,1\right]}^{n}$ that introduces solution diversity through controlled randomness.

Given a chromosome (θ,r), the decoder constructs a cover by iteratively selecting nodes in decreasing order of a composite score. At each step, the score of each candidate node *v* is computed as

(12) $$\begin{array}{cc} S(v;\theta ,r)=\; & a\cdot \deg_{U}\left(v\right)-b\cdot w\left(v\right)-c\cdot \rho \left(v\right)\\ \; & +d\cdot 1_{\mathrm{RED}}\left(v\right)+e\cdot 1_{\mathrm{GRAY}}\left(v\right)\\ \; & +0.3a\cdot \frac{\deg_{U}\left(v\right)}{\max(w\left(v\right),\epsilon_{0})}-\epsilon \cdot r_{v} \end{array}$$

where $\deg_{U}\left(v\right)$ is the uncovered degree of *v*, $\rho \left(v\right)=w\left(v\right)/\sum_{u\in N_{U}\left(v\right)}w\left(u\right)$ is the weight ratio measuring node cost relative to uncovered neighborhood weight, $1_{\mathrm{RED}}\left(v\right)$ and $1_{\mathrm{GRAY}}\left(v\right)$ are indicator functions for the respective color states, and $\epsilon_{0}=10^{-9}$ prevents division by zero.

The score function integrates six complementary objectives. The first term rewards coverage gain. The second term penalizes expensive nodes. The third term penalizes nodes with poor cost-to-neighborhood ratios. The fourth and fifth terms give priority to RED and GRAY nodes, encouraging connectivity-aware selections. The sixth term rewards coverage efficiency. The final term adds controlled random perturbation via the random key $r_{v}$. The coefficient 0.3 for the efficiency term was selected through preliminary experiments on a held-out subset of small-scale instances. Values between 0.2 and 0.5 yielded similar performance, with 0.3 providing the most consistent results across varying graph densities. Making this coefficient a separate evolvable parameter did not improve solution quality but increased the search space dimensionality. The parameter range [0,3] was chosen to encompass the natural scale of the score components. Uncovered degrees rarely exceed single digits in the tested benchmarks and the ratio term ρ(v)∈[0,1] by construction. Extending the range beyond [0,3] did not improve convergence in preliminary tests.

The decoding process works as follows. Starting with all nodes WHITE, the decoder repeatedly (1) computes the score (12) for all candidate nodes, (2) selects the highest-scoring node $v^{*}$, (3) adds $v^{*}$ to the cover and colors it BLACK, (4) colors its WHITE neighbors GRAY, (5) assigns uncovered incident edges of $v^{*}$ to it in score-consistent order until capacity is exhausted, and (6) repeats until all edges are covered. After construction, a connectivity repair adds bridge-path vertices if the cover is disconnected, and a force-cover stage handles any residual uncovered edges.

After the decoder constructs a candidate cover *C*, the fitness is evaluated with constraint violation penalties:

(13) $$f\left(C\right)=W\left(C\right)+\lambda_{U}\cdot U\left(C\right)+\lambda_{c}\left(1-I_{\mathrm{conn}}\right)+\lambda_{k}\left(1-I_{\mathrm{cap}}\right)$$

where $W\left(C\right)=\sum_{v\in C}w\left(v\right)$ is the total weight, U(C) counts uncovered edges, $I_{\mathrm{conn}}$ and $I_{\mathrm{cap}}$ are binary indicators for connectivity and capacity feasibility, respectively, and $\lambda_{U}=10^{6}$ and $\lambda_{c}=\lambda_{k}=10^{5}$ are large penalty coefficients. These magnitudes are chosen to enforce a strict lexicographic ordering among the objective components: any single uncovered edge incurs a penalty that dominates the maximum possible weight difference across all feasible solutions, and similarly, a connectivity or capacity violation outweighs any achievable weight improvement, thereby rendering infeasible solutions non-competitive regardless of their weight advantage. The relative scaling between $\lambda_{U}$ and $\lambda_{c}=\lambda_{k}$ reflects the hierarchical priority of edge coverage over structural feasibility constraints, as an uncovered edge directly violates the problem definition, whereas connectivity and capacity failures represent softer structural requirements that may be remedied through local repair operators. Reducing these coefficients by an order of magnitude was found to be insufficient in preliminary experiments, as infeasible solutions occasionally survived selection pressure and propagated through the population, ultimately degrading overall solution quality.

The initial population of size *P* is generated as follows. One chromosome is seeded with the GRCCVC solution by reverse-engineering the θ parameters that would reproduce the ratio-based greedy ordering. The remaining P−1 chromosomes are generated randomly. Parent selection uses tournament selection with selection pressure parameter $p_{s}$. In each tournament, two chromosomes are drawn uniformly at random from the population, and the one with better (lower) fitness is selected as a parent with probability $p_{s}$. Given two parent chromosomes $\left(\theta_{1},r_{1}\right)$ and $\left(\theta_{2},r_{2}\right)$, crossover produces an offspring $\left(\theta_{c},r_{c}\right)$ by independently recombining the parameter vector and the random-key vector. For the θ vector, each component $\theta_{c,i}$ is drawn uniformly from the interval $\left[\min\left(\theta_{1,i},\theta_{2,i}\right),\max\left(\theta_{1,i},\theta_{2,i}\right)\right]$. For the random-key vector, each $r_{c,v}$ is inherited from parent 1 or parent 2 with equal probability. The offspring chromosome is mutated with probability $p_{m}$. For the θ vector, each component is perturbed by adding Gaussian noise N(0,0.3), clamped to [0,3]. For the random-key vector, each key $r_{v}$ is re-drawn uniformly from [0,1] with a small per-key probability. The complete HGA procedure is described in Algorithm 3. At each generation, the algorithm either creates an offspring through selection–crossover–mutation (with probability $p_{c}$) or generates a completely random chromosome (with probability $1-p_{c}$), providing periodic injections of diversity.
**Algorithm 3:** Proposed HGA for MWCCVC.
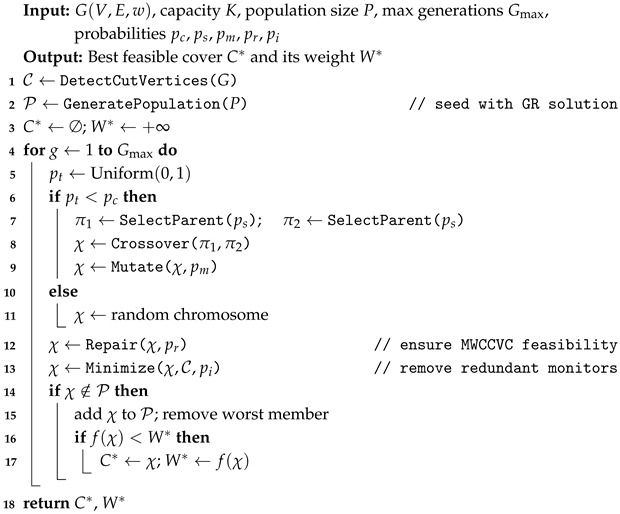


The repair procedure described in Algorithm 4 ensures that a chromosome encodes a valid MWCCVC by iteratively adding nodes until all edges are covered and the induced subgraph is connected.
**Algorithm 4:** Repair Chromosome.
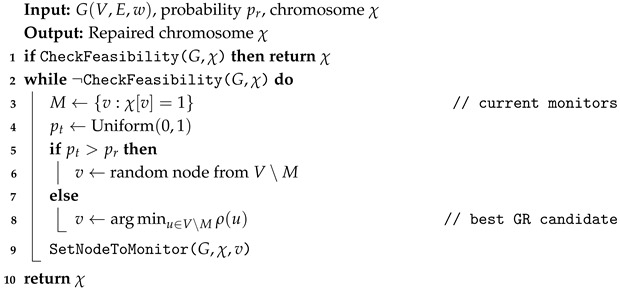


The minimize procedure presented in Algorithm 5 removes redundant monitors to reduce the total weight. A monitor *v* is deemed redundant if (i) its weight exceeds the total weight of its GRAY neighbors; (ii) it is not a black cut vertex; and (iii) it is not a global cut vertex of the original graph *G*, which is precomputed using the Hopcroft–Tarjan algorithm [63].
**Algorithm 5:** Minimize Chromosome.
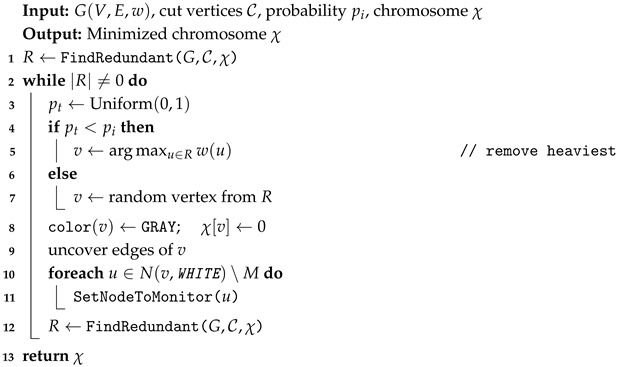


### 5.3. Enhanced HGA (HGA-v2)

HGA-v2 retains the chromosome concept and the decoder–repair–minimize pipeline but introduces five enhancements. First, the decoder uses top-*L* stochastic selection with L=3. Instead of always selecting the highest-scored candidate, it samples uniformly from the top-*L* nodes, increasing diversification and helping the search escape basins of attraction associated with greedy local optima. The value L=3 was determined through experiments on medium-scale instances where L∈{2,3,5,10}. The setting L=2 offered insufficient diversification, whereas L=5 and L=10 degraded constructive quality by selecting too many suboptimal nodes, while L=3 achieved the best balance between exploration and solution quality. Second, the penalized objective includes a cover-size tie-breaker:

(14) $$f_{2}\left(C\right)=W\left(C\right)+0.001\left|C\right|+\lambda_{U}U\left(C\right)+\lambda_{c}\left(1-I_{\mathrm{conn}}\right)+\lambda_{k}\phi_{\mathrm{cap}}\left(C\right)$$

where the 0.001|C| term favors smaller covers when weights are nearly equal. Third, the crossover is arithmetic on θ and uniform on random keys. Fourth, a feasible-only local search attempts profitable node removals and light-node swaps after decoding. Fifth, a stall threshold of 10 generations triggers early termination if no improvement is found.

For all three greedy methods, the constructive cost is O(nm). With connectivity repair, GCCVC and GRCCVC achieve $O(nm+\kappa^{2}m\log n)$ where κ is the component count before repair, while GWCCVC achieves O(nm+κ(n+m)). For HGA and HGA-v2, each generation evaluates *P* chromosomes, yielding

(15) $$T_{\mathrm{HGA}}=O\left(P\cdot G_{\max}\left(nm+\kappa^{2}m\log n+L\cdot T_{\mathrm{flow}}\left(n,m\right)\right)\right)$$

where *L* is the local search effort and $T_{\mathrm{flow}}$ is the max-flow validation cost.

## 6. Experimental Setup

The experimental study uses the benchmark dataset given in [1,53]. We evaluate three benchmark tiers. The small-scale tier contains graphs with |V|∈{10,15,20,25}, the medium-scale tier contains graphs with |V|∈{50,100,150,200}, and the large-scale tier contains graphs with |V|∈{250,500,750,1000}. In the small and medium tiers, connectivity ratios m/n∈{2,4,6,8} are used with 10 seeds per (n,m) pair, yielding 130 small and 160 medium instances. In the large tier, connectivity ratios m/n∈{2,4,6,8,10} are used with a single seed per pair, yielding 19 instances.

Six methods are compared on the small and medium tiers, namely GD, GR, GW, HGA, HGA-v2, and B&B. The configuration of the B&B solver depends on the size of the instance: for small instances (n≤25), the algorithm is executed until completion to obtain a proven optimal solution, whereas for medium-sized instances, a time limit equal to twice the longest runtime of the heuristic methods is imposed. On the large tier, five methods are evaluated, as B&B is excluded due to its computational infeasibility at scale.

For each instance, the capacity parameter *K* is determined via a binary search procedure over the feasible range of capacity values. At each step of the search, GRCCVC is executed to evaluate the resulting solution, and the corresponding vertex cover weight is computed. Among all feasible *K* values encountered during this process, the selected *K* is the one that yields the minimum total vertex cover weight under GRCCVC, while satisfying the MWCCVC feasibility constraints.

The HGA configuration employs a population size of 100 chromosomes and runs for 100 generations on the large tier. Small-scale experiments consider graphs with n∈{10,15,20,25} and connectivity ratios m/n∈{2,4,6,8,10} over multiple random seeds. Medium-scale experiments use graphs with n∈{50,100,150,200} under the same connectivity settings. The large-tier experiments consider graphs with n∈{250,500,750,1000}, with one seed per configuration and capacity values ranging from K=7 to K=36. The simulation parameters and benchmark characteristics for all experimental tiers are reported in Table 2.

## 7. Results and Evaluation

Table 3, Table 4 and Table 5 summarize the overall performance across all three benchmark tiers using standardized columns, namely average total weight (Weight), average cover size (|C|), average execution time (Time), weight gap relative to HGA (Gap), and feasibility rate. In particular, Gap (%) denotes the percentage deviation of the weight value obtained by each method with respect to HGA, which is taken as the reference solution. Accordingly, it is computed by expressing the difference between the weight value of the corresponding method and that of HGA as a percentage of the HGA value. All methods achieve 100% feasibility across all 309 tested instances.

### 7.1. Small-Scale Results

The most striking result on the 130 small-scale instances is that HGA exactly matches the B&B solver on every graph, yielding a gap of 0.00%, as shown in Table 3. HGA achieves the B&B-optimal weight on all 130 instances, recording 130 wins and no ties or losses against each greedy method, which makes statistical testing unnecessary at this scale. HGA-v2 is also highly competitive with an average gap of only 0.06%, while being about 11× faster than HGA. Among the constructive heuristics, GR is clearly the strongest baseline with a gap of 5.99%, while GW consistently produces the heaviest solutions with a gap of 22.19%.

### 7.2. Medium-Scale Results

The medium-scale experiments expose the practical breakdown point of exact optimization. The B&B solver times out on all 160 instances and cannot provide proven optima. HGA achieves the best result on every medium-scale graph and improves on the time-limited B&B solutions by 6.3% on average, as declared in Table 4. HGA-v2 provides the strongest quality–speed compromise, with a gap of 4.6% above HGA while being roughly an order of magnitude faster. GR remains the strongest greedy baseline with a 7.1% gap at sub-40 ms runtime.

To assess statistical significance, we apply the Wilcoxon signed-rank test to the 160 paired weight differences between HGA and each competing method. All pairwise comparisons yield p<0.001, confirming that the weight improvements of HGA over GD, GR, GW, HGA-v2, and B&B are statistically significant at the 1% level. Among the greedy methods, GR is significantly better than GD with p<0.001 and also significantly better than GW with p<0.001.

### 7.3. Large-Scale Results

The large-scale experiments consider graphs with |V|∈{250,500,750,1000} and include 19 instances, forming the primary scalability test. B&B is excluded due to computational infeasibility. HGA maintains its quality leadership on all 19 instances, achieving a 100% win rate. However, the computational cost becomes substantial. HGA requires on average 33 min per instance, and the most demanding graph with *n* = 1000 and *m* = 8000 requires approximately 2 h, as shown in Table 5, while a breakdown of average total weight by node count is further provided in Table 6.

HGA-v2 emerges as the most practical method at this scale with only a 3.1% gap and an average runtime of 3.1 min, which is approximately 10× faster than HGA. Among greedy methods, GR continues to perform well with a 4.3% gap and sub-second runtime. With 19 instances and a single seed per configuration, formal statistical tests have limited power at this scale. Instead, we report instance-level dominance where HGA achieves the lowest weight on 19/19 instances against every competitor. HGA-v2 wins on 17/19 instances against GR, with GR prevailing on two dense graphs at |V|=1000. These consistent dominance patterns, combined with the statistically validated medium-scale results, support the generalizability of the method ranking.

### 7.4. Cross-Scale Analysis

**Figure 3:**
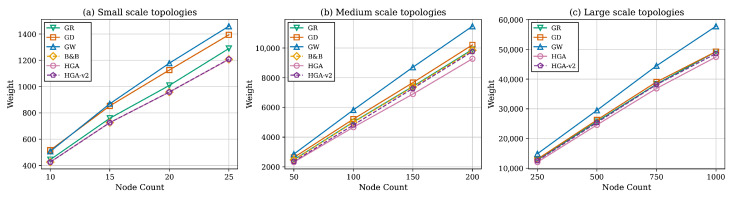
Comparison of total weight values with node count.

**Figure 4:**
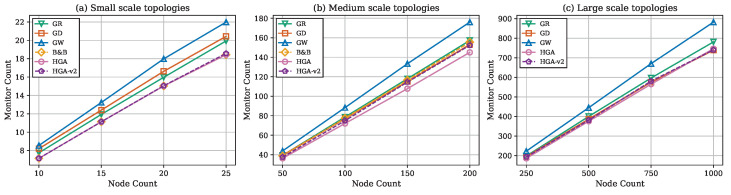
Cover set size against total node count.

**Figure 5:**
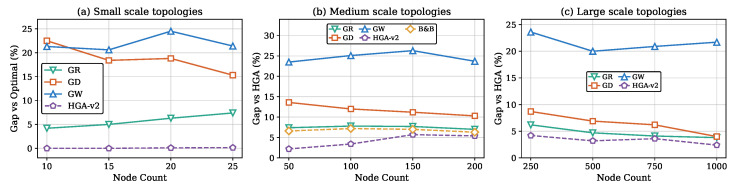
Weight gap relative to HGA against node count.

**Figure 6:**
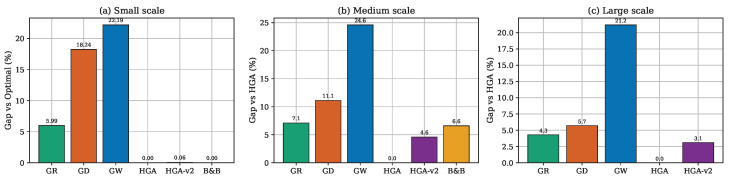
Overall weight gap relative to HGA across all three benchmark tiers.

Figure 3 presents total weight against node count across all three scales. HGA consistently produces the lowest weight at every node count. Figure 4 shows monitor count (cover size) trends, confirming that methods producing smaller covers consistently achieve lower total weights. Figure 5 reports the gap relative to HGA; the method ranking GW > GD > GR > HGA-v2 is preserved across all scales. Figure 6 provides the aggregate gap comparison.

### 7.5. Impact of Connectivity Ratio (m/n)

**Figure 7:**
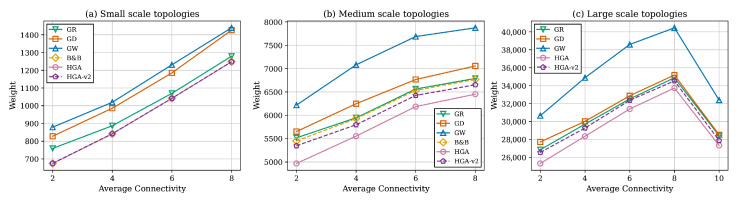
Total weight values against connectivity.

Table 7 and Table 8 and Figure 7 summarize the effect of graph density. A clear monotonic trend appears with the average weight increasing as connectivity grows for all methods. HGA remains best at every density level across both scales. The relative gaps narrow as graphs become denser.

### 7.6. Runtime Analysis

**Figure 8:**
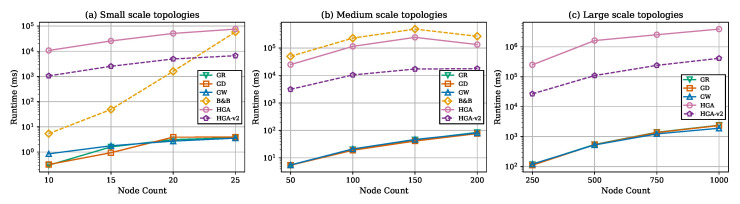
Runtime comparison (log scale) across all three benchmark tiers.

Figure 8 compares runtimes on a logarithmic scale across all three benchmark tiers. Greedy heuristics remain in the millisecond-to-second regime even at |V|=1000. HGA’s runtime increases from seconds (small) to minutes (medium) to tens of minutes (large), with worst case approaching 2 h at |V|=1000. HGA-v2 consistently maintains ∼10× speedup over HGA. The B&B solver is practical only at small scale.

## 8. Discussion

A clear transition emerges across the three benchmark tiers. At small scale (n=10–25), the B&B solver provides optimal references, and HGA matches optimality on 100% of instances. At medium scale (n=50–200), B&B degrades to 100% timeout, and HGA surpasses it by the margin reported in Table 4. At large scale (n=250–1000), B&B is entirely excluded, and HGA remains the quality leader at the cost of substantial computation. Since HGA is proven optimal at small scale, its consistent superiority at medium and large scales strongly suggests near-optimal performance throughout.

The method ranking HGA > HGA-v2 > GR > GD > GW remains perfectly consistent across all three scales, as illustrated in Figure 6, providing strong evidence of generalizability. One notable observation at large scale is the convergence of GR and GD at |V|=1000, as reported in Table 6. This convergence can be attributed to the diminishing discriminative power of the ratio criterion in dense graphs. As m/n increases, most nodes have similar neighborhood weight sums, causing ρ(v) values to cluster in a narrow range. Consequently, the ratio-based ranking approaches the degree-based ranking, and both heuristics select similar node sequences. This suggests that for very large, dense networks, simpler degree-based criteria may be sufficient, reducing implementation complexity without meaningful quality loss.

The effectiveness of HGA-v2’s top-*L* stochastic selection stems from two complementary mechanisms. First, it prevents the decoder from deterministically reproducing the same greedy sequence for chromosomes with similar θ vectors, thereby increasing effective population diversity. Second, it enables the search to escape greedy construction basins where the globally highest-scoring node at each step leads to a locally optimal but globally suboptimal cover. The early-termination stall detector complements this by reallocating computation from stagnant runs to fresh restarts.

Beyond the time budget-based selection, network characteristics also inform method choice. For sparse networks with m/n≤4, where the quality gaps between methods are largest, as reported in Table 7, investing in HGA yields the greatest benefit. For dense networks with m/n≥8, where all methods converge in quality, GR’s sub-second runtime makes it the pragmatic choice. In heterogeneous networks where node weights vary widely, ratio-based methods (GR and HGA) significantly outperform degree-only selection (GD), since weight-unaware node choices can inadvertently select high-cost monitors.

Three distinct operating regimes emerge for practitioners. For applications requiring the highest solution quality, HGA is recommended, achieving a 0% gap at small scale and delivering the best-known solutions at medium and large scales, although with runtimes ranging from minutes to hours. For scenarios requiring a balance between quality and speed, HGA-v2 offers an attractive compromise with a gap of at most 3.1% while being approximately 10× faster, typically completing within seconds to minutes. For real-time applications, GR provides the most practical option, maintaining a gap of at most 7% with sub-second execution across all scales.

## 9. Conclusions

In this article, we formulated the MWCCVC problem for energy-efficient link monitoring in IoT-enabled WSNs. We proposed five solution methods, namely three greedy heuristics (GD, GR, GW) and two hybrid genetic algorithms (HGA, HGA-v2) and also developed a B&B reference solver. All proposed methods enforce full edge coverage, induced-subgraph connectivity, and capacity feasibility, while the B&B solver provides provably optimal solutions through LP relaxation bounds and constraint-based pruning strategies.

Extensive experiments on 130 small-scale, 160 medium-scale, and 19 large-scale benchmark instances reveal several key findings. HGA achieves provably optimal solutions at small scale and maintains quality leadership across all tested sizes, as reported in Table 3, Table 4 and Table 5. On medium-scale instances, HGA consistently outperforms the time-limited B&B solver, with statistically significant improvements confirmed by Wilcoxon signed-rank tests yielding p<0.001. On large-scale instances with up to 1000 nodes, HGA remains the best performer on every graph, although at significant computational cost. HGA-v2 provides an exceptional quality–speed trade-off across all scales, and GR achieves solution quality comparable to the B&B solver while running orders of magnitude faster. The performance ordering HGA > HGA-v2 > GR > GD > GW remains consistent across all three benchmark tiers.

Future work will pursue several complementary directions. First, systematically adapting existing state-of-the-art metaheuristics to the MWCCVC formulation constitutes a substantial research direction. A rigorous comparative study against the proposed HGA requires problem-specific encoding schemes and constraint-handling mechanisms tailored to each metaheuristic; therefore, this effort has been explicitly deferred to future work. Second, distributed MWCCVC algorithms suitable for in-network execution will be investigated, enabling backbone construction without centralized coordination. Third, adaptive capacity assignment strategies will be explored to address heterogeneous and dynamically changing node capabilities in practical IoT deployments. Fourth, parallel genetic operators will be developed to improve the scalability of HGA for very large network instances, where sequential evaluation of fitness and feasibility becomes a bottleneck. Finally, the proposed framework will be validated on physical IoT testbeds to assess its robustness under real-world conditions, including link asymmetry, packet loss, and hardware heterogeneity.

## Figures and Tables

**Table 2:** Simulation parameters and benchmark characteristics.

Parameter	Small	Medium	Large
Node counts	{10,15,20,25}	{50,100,150,200}	{250,500,750,1000}
Connectivity ratio m/n	{2,4,6,8}	{2,4,6,8}	{2,4,6,8,10}
Seeds per (n,m)	10	10	1
Total instances	130	160	19
Methods	5 + B&B	5 + B&B	5 (no B&B)
Capacity *K*	3–18 (avg. 9.0)	4–30 (avg. 14.2)	7–36 (avg. 19.1)
HGA pop./gen.	40/60	40/60	100/100
B&B budget	Sufficient	2× longest heur.	N/A
Validity	100%	100%	100%

**Table 3:** Small-scale performance summary (130 instances). Gap is relative to HGA (%).

Method	Weight	|*C*|	Time (s)	Gap (%)	Valid
GD	1051.2	15.5	0.003	+18.24	130/130
GR	950.4	15.0	0.003	+5.99	130/130
GW	1089.8	16.7	0.003	+22.19	130/130
HGA	898.9	14.0	46.6	0.00	130/130
HGA-v2	899.6	14.0	4.3	+0.06	130/130
B&B	898.9	14.0	18.5	0.00	130/130

**Table 4:** Medium-scale performance summary (160 instances). Gap is relative to HGA (%).

Method	Weight	|*C*|	Time (s)	Gap (%)	Valid
GD	6430	96.1	0.04	+11.1	160/160
GR	6204	98.3	0.04	+7.1	160/160
GW	7215	110.4	0.04	+24.6	160/160
HGA	5790	90.3	130.1	—	160/160
HGA-v2	6056	94.8	12.2	+4.6	160/160
B&B *	6170	97.0	260.3	+6.6	160/160

* B&B times out on all 160 medium instances; values are best found within the time limit.

**Table 5:** Large-scale performance summary (19 instances). Gap is relative to HGA (%).

Method	Weight	|*C*|	Time (s)	Gap (%)	Valid
GD	30,989	460.1	1.0	+5.7	19/19
GR	30,583	478.7	1.0	+4.3	19/19
GW	35,553	537.7	0.9	+21.2	19/19
HGA	29,330	453.6	1981	—	19/19
HGA-v2	30,241	459.8	186	+3.1	19/19

B&B is excluded at large scale due to computational infeasibility.

**Table 6:** Large-scale average total weight versus node count |V|.

|*V*|	GD	GR	GW	HGA	HGA-v2	Inst.
250	13,068	12,793	14,878	12,062	12,545	5
500	26,244	25,731	29,505	24,581	25,375	5
750	39,038	38,291	44,479	36,844	38,133	5
1000	49,261	49,249	57,798	47,458	48,582	4

**Table 7:** Medium-scale average total weight versus connectivity ratio m/n.

*m*/*n*	GD	GR	GW	HGA	HGA-v2	B&B
2	5652	5518	6218	4968	5348	5450
4	6247	5946	7081	5554	5797	5929
6	6766	6563	7686	6187	6426	6532
8	7056	6789	7873	6452	6653	6768

**Table 8:** Large-scale average total weight versus connectivity ratio m/n.

*m*/*n*	GD	GR	GW	HGA	HGA-v2
2	27,724	26,855	30,653	25,322	26,552
4	30,022	29,711	34,887	28,359	29,278
6	32,874	32,517	38,594	31,400	32,365
8	35,188	34,876	40,451	33,739	34,536
10	28,521	28,412	32,387	27,330	27,889

## Data Availability

The implementation of the algorithms and benchmark instances used in this work are publicly available at: https://github.com/zadlabege/MWCCVC (accessed on 15 March 2026).

## Abbreviations

The following abbreviations are used in this manuscript:

B&B	Branch and Bound
BFS	Breadth-First Search
CapVC	Capacitated Vertex Cover
CDS	Connected Dominating Set
CRO	Chemical Reaction Optimization
CVC	Connected Vertex Cover
GA	Genetic Algorithm
GCCVC	Greedy Degree Connected Capacitated Vertex Cover
GD	Greedy Degree
GR	Greedy Ratio
GRASP	Greedy Randomized Adaptive Search Procedure
GRCCVC	Greedy Ratio Connected Capacitated Vertex Cover
GW	Greedy Weight
GWCCVC	Greedy Weight Connected Capacitated Vertex Cover
HGA	Hybrid Genetic Algorithm
HGA-v2	Enhanced Hybrid Genetic Algorithm
IoT	Internet of Things
MS-ITS	Multi-Start Iterative Tabu Search
LP	Linear Programming
MVC	Minimum Vertex Cover
MWCCVC	Minimum Weighted Connected Capacitated Vertex Cover
MWCVC	Minimum Weighted Connected Vertex Cover
MWVC	Minimum Weighted Vertex Cover
NSGA	Nondominated Sorting Genetic Algorithm
PBIG	Population Based Iterated Greedy
PSO	Particle Swarm Optimization
PTAS	Polynomial-Time Approximation Scheme
SA	Simulated Annealing
UDG	Unit Disk Graph
VC	Vertex Cover
WCapVC	Weighted Capacitated Vertex Cover
WCVC	Weighted Connected Vertex Cover
WSN	Wireless Sensor Network

## References

[1] Akusta Dagdeviren Z.A. (2021). Weighted connected vertex cover based energy-efficient link monitoring for wireless sensor networks towards secure Internet of Things. IEEE Access.

[2] Shafi M., Jha R.K., Jain S. (2023). LGTBIDS: Layer-Wise Graph Theory-Based Intrusion Detection System in Beyond 5G. IEEE Trans. Netw. Serv. Manag..

[3] Alwasel B., Salim A., Khedr A.M., Osamy W. (2024). Dominating Sets-Based Approach for Maximizing Lifetime of IoT-Based Heterogeneous WSNs Enabled Sustainable Smart City Applications. IEEE Access.

[4] Zanjireh M.M., Larijani H. A survey on centralised and distributed clustering routing algorithms for WSNs. Proceedings of the IEEE Vehicular Technology Conference (VTC Spring).

[5] Akusta Dagdeviren Z. (2020). Vertex cover based link monitoring techniques for wireless sensor networks. Selcuk. Univ. J. Eng. Sci..

[6] Godquin T., Barbier M., Gaber C., Grimault J.L., Le Bars J.M. (2020). Applied graph theory to security: A qualitative placement of security solutions within IoT networks. J. Inf. Secur. Appl..

[7] Borndörfer R., Schwartz S., Surau W. (2023). Vertex covering with capacitated trees. Networks.

[8] Kepner J., Aaltonen P., Bader D.A., Buluç A., Franchetti F., Gilbert J.R., Hutchison D., Kumar M., Lumsdaine A., Meyerhenke H. Mathematical foundations of the GraphBLAS. Proceedings of the IEEE High Performance Extreme Computing Conference (HPEC).

[9] Hassan J.A., Laja L.S. (2024). Vertex cover zero forcing sets in graphs. Comput. Sci..

[10] Yakut S., Öztemiz F., Karci A. (2023). A new robust approach to solve minimum vertex cover problem: Malatya vertex-cover algorithm. J. Supercomput..

[11] Cai S., Su K., Luo C., Sattar A. (2013). NuMVC: An efficient local search algorithm for minimum vertex cover. J. Artif. Intell. Res..

[12] Ma Z., Fan Y., Su K., Li C., Sattar A. (2016). Local search with noisy strategy for minimum vertex cover in massive graphs. PRICAI 2016: Trends in Artificial Intelligence.

[13] Witt C. (2012). Analysis of an iterated local search algorithm for vertex cover in sparse random graphs. Theor. Comput. Sci..

[14] Ugurlu O. New heuristic algorithm for unweighted minimum vertex cover. Proceedings of the Panhellenic Conference on Informatics (PCI).

[15] Wu J., Shen X., Jiao K. (2019). Game-based memetic algorithm to the vertex cover of networks. IEEE Trans. Cybern..

[16] Lazzarinetti G., Dondi R., Manzoni S., Zoppis I. (2024). An Attention-Based Method for the Minimum Vertex Cover Problem on Complex Networks. Algorithms.

[17] Ung E., Kao M.J. (2022). Approximation Algorithm for Vertex Cover with Multiple Covering Constraints. Algorithmica.

[18] Cheung C.W., Goemans M.X., Wong S. Improved algorithms for vertex cover with hard capacities on multigraphs and hypergraphs. Proceedings of the 25th Annual ACM-SIAM Symposium on Discrete Algorithms (SODA).

[19] Caskurlu B., Mkrtchyan V., Parekh O., Subramani K. (2014). On partial vertex cover and budgeted maximum coverage problems in bipartite graphs. Theoretical Computer Science.

[20] Bazzi A., Fiorini S., Pokutta S., Svensson O. No small linear program approximates vertex cover within a factor 2 − *ε*. Proceedings of the 56th IEEE Symposium on Foundations of Computer Science (FOCS).

[21] Kavalci V., Ural A., Dagdeviren O. (2014). Distributed vertex cover algorithms for wireless sensor networks. Int. J. Comput. Netw. Commun..

[22] Yigit Y., Ileri C.U., Dagdeviren O. Fault tolerance performance of self-stabilizing independent set algorithms on a covering-based problem: The case of link monitoring in WSNs. Proceedings of the 2018 5th International Conference on Electrical and Electronic Engineering (ICEEE).

[23] Akram V.K., Ugurlu O. (2022). A localized distributed algorithm for vertex cover problem. J. Comput. Sci..

[24] Xu H., Kumar T.K.S., Koenig S. (2016). A new solver for the minimum weighted vertex cover problem. Integration of AI and OR Techniques in Constraint Programming.

[25] Li R., Hu S., Cai S., Gao J., Wang Y., Yin M. (2020). NuMWVC: A novel local search for minimum weighted vertex cover problem. J. Oper. Res. Soc..

[26] Cai S., Li Y., Hou W., Wang H. (2019). Towards faster local search for minimum weight vertex cover on massive graphs. Inf. Sci..

[27] Zhou T., Lü Z., Wang Y., Hao J.K. (2016). Multi-start iterated tabu search for the minimum weight vertex cover problem. J. Comb. Optim..

[28] Jovanovic R., Sanfilippo A.P., Voß S. (2022). Fixed set search applied to the multi-objective minimum weighted vertex cover problem. J. Heuristics.

[29] Langedal K., Langguth J., Manne F., Schroeder D.T., Schulz C., Uçar B. (2022). Efficient Minimum Weight Vertex Cover Heuristics Using Graph Neural Networks. 20th International Symposium on Experimental Algorithms (SEA 2022).

[30] Li Y., Yang Z., Wang W. (2017). Complexity and algorithms for the connected vertex cover problem in 4-regular graphs. Appl. Math. Comput..

[31] Zhang Y., Wu J., Zhang L., Zhao P., Zhou J., Yin M. (2018). An Efficient Heuristic Algorithm for Solving Connected Vertex Cover Problem. Math. Probl. Eng..

[32] Fan L., Zhang Z., Wang W. (2011). PTAS for minimum weighted connected vertex cover problem with c-local condition in unit disk graphs. J. Comb. Optim..

[33] Akusta Dagdeviren Z.A. (2023). A metaheuristic algorithm for vertex cover based link monitoring and backbone formation in wireless ad hoc networks. Expert Syst. Appl..

[34] Yigit Y., Dagdeviren Z.A., Dagdeviren O., Challenger M. Performance Evaluation of Capacitated Vertex Cover Algorithms for Security Applications in Wireless Sensor Networks. Proceedings of the 2021 7th International Conference on Electrical, Electronics and Information Engineering (ICEEIE).

[35] Yigit Y., Dagdeviren O., Challenger M. (2022). Self-stabilizing capacitated vertex cover algorithms for Internet-of-Things-enabled wireless sensor networks. Sensors.

[36] Wei H.T., Hon W.K., Horn P., Liao C.S., Sadakane K. (2022). Approximating Dynamic Weighted Vertex Cover with Soft Capacities. Algorithmica.

[37] Chu H., Lin B., Iwata S., Kakimura N. (2023). FPT Approximation Using Treewidth: Capacitated Vertex Cover, Target Set Selection and Vector Dominating Set. 34th International Symposium on Algorithms and Computation (ISAAC 2023).

[38] Mishra R., Jha S.K., Kshetri N., Bhusal B., Rahman M.M., Rana M.M., Eli A.A., Islam K.A., Pokharel B.P. (2025). nodeWSNsec: A hybrid metaheuristic approach for reliable security and node deployment in wireless sensor networks. Int. J. Adv. Comput. Sci. Appl..

[39] Hilda S., Kalaiselvi C. (2025). Multi agent energy management prediction and load balanced clustering framework for WSNs using quantum bio-inspired PSO optimization. Indian J. Sci. Technol..

[40] Anusuya P., Vanitha C.N., Cho J., Veerappampalayam Easwaramoorthy S. (2024). A comprehensive review of sensor node deployment strategies for maximized coverage and energy efficiency in wireless sensor networks. PeerJ Comput. Sci..

[41] Yigit Y., Akram V.K., Dagdeviren O. (2021). Breadth-first search tree integrated vertex cover algorithms for link monitoring and routing in wireless sensor networks. Comput. Netw..

[42] Quan C., Guo P. (2021). A local search method based on edge age strategy for minimum vertex cover problem in massive graphs. Expert Syst. Appl..

[43] Zhang Y., Wang S., Liu C., Zhu E. (2023). TIVC: An Efficient Local Search Algorithm for Minimum Vertex Cover in Large Graphs. Sensors.

[44] Guo P., Quan C., Chen H. (2019). MEAMVC: A Membrane Evolutionary Algorithm for Solving Minimum Vertex Cover Problem. IEEE Access.

[45] Khattab H., Mahafzah B.A., Sharieh A. (2022). A hybrid algorithm based on modified chemical reaction optimization and best-first search algorithm for solving minimum vertex cover problem. Neural Comput. Appl..

[46] Shimizu S., Yamaguchi K., Saitoh T., Masuda S. A fast heuristic for the minimum weight vertex cover problem. Proceedings of the 2016 IEEE/ACIS 15th International Conference on Computer and Information Science (ICIS).

[47] Cai S., Hou W., Lin J., Li Y. Improving local search for minimum weight vertex cover by dynamic strategies. Proceedings of the 27th International Joint Conference on Artificial Intelligence (IJCAI).

[48] Islam M.R., Arif I.H., Shuvo R.H. (2019). Generalized vertex cover using chemical reaction optimization. Appl. Intell..

[49] Pourhassan M., Friedrich T., Neumann F. On the use of the dual formulation for minimum weighted vertex cover in evolutionary algorithms. Proceedings of the ACM/SIGEVO Conference on Foundations of Genetic Algorithms (FOGA).

[50] Krithika R., Majumdar D., Raman V. (2018). Revisiting Connected Vertex Cover: FPT Algorithms and Lossy Kernels. Theory Comput. Syst..

[51] Guha S., Khuller S. (1998). Approximation Algorithms for Connected Dominating Sets. Algorithmica.

[52] Du D.Z., Graham R., Pardalos P., Wu W. (2012). Approximation for minimum 2-connected *p*-dominating set. Proceedings of the International Conference on Combinatorial Optimization and Applications (COCOA).

[53] Akusta Dagdeviren Z.A., Aydin D., Cinsdikici M. (2017). Two population-based optimization algorithms for minimum weight connected dominating set problem. Appl. Soft Comput..

[54] Potluri A., Singh A. (2013). Hybrid metaheuristic algorithms for minimum weight dominating set. Appl. Soft Comput..

[55] Dahmri H., Bouamama S., Chikhi S., Amine A., Chaoui A., Saidouni D., Kholladi M. (2021). Improved NSGA-II for Minimum Weight Minimum Connected Dominating Set Problem. Modelling and Implementation of Complex Systems.

[56] Wang L., Zhang X., Zhang Z., Broersma H. (2015). A PTAS for the minimum weight connected vertex cover *P*_3_ problem on unit disk graphs. Theor. Comput. Sci..

[57] Sharif A., Potdar V., Chang E. Wireless multimedia sensor network technology: A survey. Proceedings of the 7th IEEE International Conference on Industrial Informatics.

[58] Saba T., Rehman A., Haseeb K., Bahaj S.A., Jeon G. (2022). Energy-efficient edge optimization embedded system using graph theory with 2-tiered security. Electronics.

[59] Bar-Yehuda R., Flysher G., Mestre J., Rawitz D. Approximation of partial capacitated vertex cover. Proceedings of the 15th European Symposium on Algorithms (ESA).

[60] Guha S., Hassin R., Khuller S., Or E. (2003). Capacitated vertex covering. J. Algorithms.

[61] Delbot F., Laforest C., Rovedakis S., Aguilera M.K., Querzoni L., Shapiro M. (2014). Self-stabilizing Algorithms for Connected Vertex Cover and Clique Decomposition Problems. Principles of Distributed Systems.

[62] Karp R.M. (2010). Reducibility among combinatorial problems. 50 Years of Integer Programming 1958–2008.

[63] Hopcroft J., Tarjan R. (1973). Algorithm 447: Efficient algorithms for graph manipulation. Commun. ACM.

